# Yeast Endocytic Adaptor AP-2 Binds the Stress Sensor Mid2 and Functions in Polarized Cell Responses

**DOI:** 10.1111/tra.12155

**Published:** 2014-02-25

**Authors:** Bernardo Chapa-y-Lazo, Ellen G Allwood, Iwona I Smaczynska-de Rooij, Mary L Snape, Kathryn R Ayscough

**Affiliations:** Department of Biomedical Science, University of SheffieldSheffield, S10 2TN, UK

**Keywords:** *Candida albicans*, endocytosis, Mid2, pheromone, polarity, pseudohyphae, *Saccharomyces cerevisiae*, stress

## Abstract

The AP-2 complex is a heterotetrameric endocytic cargo-binding adaptor that facilitates uptake of membrane proteins during mammalian clathrin-mediated endocytosis. While budding yeast has clear homologues of all four AP-2 subunits which form a complex and localize to endocytic sites *in vivo*, the function of yeast AP-2 has remained enigmatic. Here, we demonstrate that AP-2 is required for hyphal growth in *Candida albicans* and polarized cell responses in *Saccharomyces cerevisiae*. Deletion of *APM4*, the cargo-binding mu subunit of AP-2, causes defects in pseudohyphal growth, generation of a mating projection and the cell wall damage response. In an *apm4* null mutant, the cell wall stress sensor Mid2 is unable to relocalize to the tip of a mating projection following pheromone addition, or to the mother bud neck in response to cell wall damage. A direct binding interaction between Mid2 and the mu homology domain of Apm4 further supports a model in which AP-2 binds Mid2 to facilitate its internalization and relocalization in response to specific signals. Thus, Mid2 is the first cargo for AP-2 identified in yeast. We propose that endocytic recycling of Mid2 and other components is required for polarized cell responses ensuring cell wall deposition and is tightly monitored during cell growth.

AP-2 is an extremely well characterized mammalian endocytic adaptor protein linking integral membrane cargo proteins such as receptors, to the clathrin coat of vesicles forming at the plasma membrane. It is a complex of four non-identical proteins; the large (∼100 kDa) α and β subunits, the 50 kDa mu2 subunit and a smaller (17 kDa) σ2 subunit 1,2. Within the complex the mu2 and σ2 subunits are responsible for binding cargo through defined motifs. mu2 binds motifs of the type YXXΦ while σ2 binds to D/EXXXLL/I motifs 3,4. The entire complex has been the focus of structural analysis, and both closed, unlatched and open conformations of the complex have been elucidated 1,5,6. Activation to the open, cargo-binding form is associated with binding to membranes, initially through patches of basic amino acids on the α and β subunits. YXXΦ signals are bound after membrane binding and this cargo binding is proposed to be stabilized by mu subunit phosphorylation 7,8.

Targeted disruption of AP-2 by RNAi is lethal in *Caenorhabditis elegans* indicating its central importance in the endocytic process 9. More specifically, knockdown of AP-2 by siRNA causes defects in uptake of cargoes such as transferrin 10. Furthermore, AP-2 appears to play an essential role in the formation of clathrin coated vesicles since its depletion causes a highly marked reduction in the number of clathrin coated pits that form at the plasma membrane 10,11. Given the highly conserved nature of much of the endocytic machinery, including the presence of all 4 AP-2 components, it was unexpected when deletion of genes encoding AP-2 subunits in *Saccharomyces cerevisiae* did not significantly impact the function of the core endocytic machinery, nor did deletions affect uptake of either a lipophilic dye (FM4-64) or of the pheromone alpha factor 12. AP-2 components in yeast do however form a tetrameric complex 13 which colocalizes with the endocytic machinery 12. In addition, deletion of any subunit of the AP-2 complex renders cells resistant to the effect of the killer toxin K28 and prevents accumulation of the toxin inside cells 12. The mechanism of this resistance, however, is not understood as the receptor is not known, and binding to mannose residues has been shown to be critical at least for initial binding 14–16. Indeed, resistance to K28 was found on deletion of many genes encoding components involved in generating GPI anchored tails suggesting that, like the toxin K1, the receptor itself may be GPI anchored and would therefore not have a cytoplasmic tail available for direct AP-2 binding 12,17. Localization of AP-2 with the endocytic machinery, but not being essential for general functioning of the overall endocytic process, has led to the proposal that AP-2 functions as a cargo-specific adaptor.

## Results

### AP2 complex is involved in polarized hyphal growth in *C. albicans*

Despite having an AP-2 complex present in *S. cerevisiae* there has been little evidence for a function of the complex in normal cell responses. *In silico* analysis of AP-2 subunit knockout phenotypes was used to provide possible insight into functional roles of AP-2 in fungi. Interestingly, the three AP-2 gene deletions reported in *Neurospora crassa* (alpha, beta and mu subunits) all resulted in an abnormal growth pattern on plates 18. In *C. albicans*, a homozygous deletion of the gene encoding the β-adaptin affected colony morphology and there was a filamentous growth defect, while a heterozygous deletion of the mu subunit caused a defect in invasive growth 19. Taken together the data suggested a possible role for this putative endocytic adaptor in cell shape and polarized growth.

**Figure 1:**
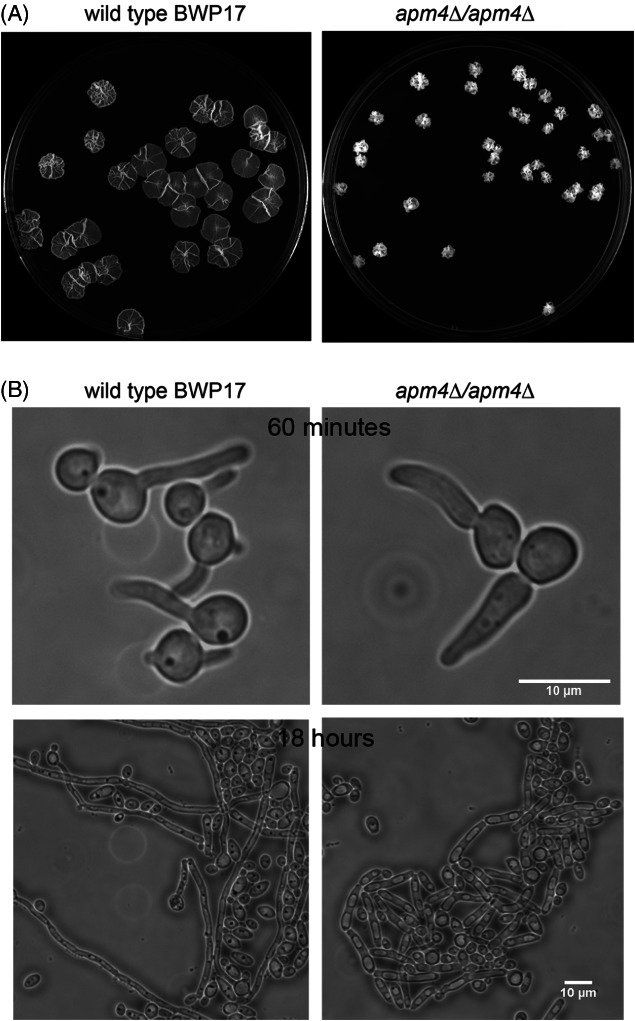
**Apm4 is required for maintenance of hyphal morphology in *Candida albicans*.** A) Wild type (BWP17) and a stain lacking both copies of *apm4* (KAY1776) were grown on Spider medium for 6 days. B) Wild type and *apm4*Δ cells were induced to form hyphae in liquid medium and the extent of hyphal formation, and morphology of resulting hyphae were observed after 60 min and 24 h. Scale bar = 10 µm.

In order to investigate the role of AP-2 in fungal growth further, we generated the homozygous deletion of the *C. albicans APM4* gene, which encodes the AP-2 mu subunit. *C. albicans* cells grow as both yeast and hyphal forms and it is proposed that endocytosis of certain cargoes may be responsible for limiting hyphal growth to the tip region 20. Growth of the mutant cells was analysed in liquid and on solid media. Deletion of *APM4* caused a marked reduction in the ability of the *C. albicans* mutants to undergo normal hyphal growth. Colonies that formed on a solid medium were able to penetrate agar but did not undergo extensive spreading (Figure 1A). The colony diameter of wild type *Candida* was more than three times greater, than that of *apm4*Δ cells. In liquid, wild type cells underwent a yeast-hyphal transition and clear hyphae were observed after 60 min. *APM4* null cells underwent morphological changes but these changes were more similar to formation of pseudohyphae, with the filaments formed being wider (hyphal diameter mean of 3.15 µm compared to 2.6 µm in wild type cells), shorter, less straight and showing constriction points when compared to wild type hyphae (Figure 1B). These data support the idea that the AP-2 complex potentially functions in maintaining, rather than establishing, sites of polarized growth in *C. albicans*.

### AP-2 is required for polarized growth in *S. cerevisiae*

**Figure 2:**
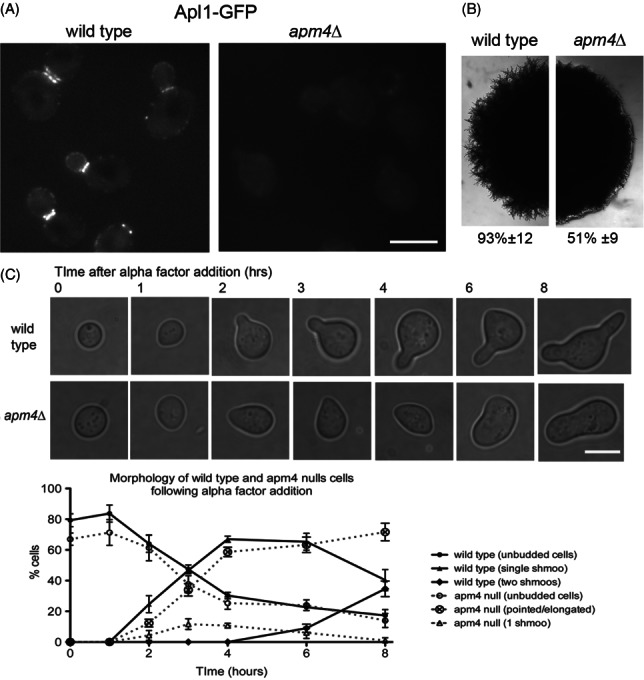
**Apm4 is required for polarized growth responses in** ***Saccharomyces cerevisiae.*** A) Cells expressing Apl1-GFP were grown to log phase and imaged as described in wild type (KAY1742) and *apm4*Δ (KAY1747) cells. Arrowheads indicate localization to endocytic patches; arrows show mother bud neck localization. Scale bar = 5 µm. B) Colony morphology of wild type and *apm4*Δ diploid cells (KAY1217 and KAY1732) was analysed on media that induces formation of pseudohyphae. C) Morphologies and quantitation of wild type and *apm4*Δ cells following addition of alpha factor for 8 h. Scale bar = 5 µm.

Using the information gained from analysis in *C. albicans*, polarized cell responses were analysed in *S. cerevisiae*. A deletion of the putative cargo-binding mu subunit (*apm4*) was generated and perturbation of the whole AP-2 complex in this mutant was confirmed using localization of the β-subunit (Apl1-GFP) as a reporter (Figure 2A).

As expected from the lack of reported phenotypes, budding appeared generally normal. However, closer analysis of the bipolar budding phenotype in diploid cells did reveal subtle changes, with an increase from 0% to 15–20% of cells showing a random budding pattern. Such a defect has previously been shown for mutations in proteins functioning at endocytic sites adding support to a function for AP-2 in endocytosis 21. Another pathway that triggers changes in polarity of *S. cerevisae* growth is initiated by changes in the nutritional environment. In conditions of limiting nitrogen but abundant carbon source, *S. cerevisiae* cells are able to undergo pseudohyphal growth. This pathway requires diploid cells to restrict growth to one end of the cell (unipolar) and to become more elongated 22. *APM4* was deleted in *S. cerevisiae* strains capable of undergoing pseudohyphal growth (Σ1278 background). Cells were analysed after 6 days on appropriate medium. As shown, while many of the *apm4*Δ cells are able to become filamentous, the extent of filamentation was reduced and the overall effect at the level of roughness of colony edges was significantly decreased (Figure 2B).

The best characterized polarized response pathway in *S. cerevisiae* is that triggered following pheromone addition 23. In response to alpha factor, cells of the MAT**a** mating type undergo morphological changes to generate a mating projection or shmoo. In the absence of *APM4* shmoo formation was severely inhibited, and appropriately shaped mating projections were not observed (Figure 2C). Projections that formed were much wider than in wild type cells suggesting a reduced ability either to direct growth or to maintain polarity at the growth site. Analysis of the proportion of wild type cells with clearly defined projections was followed over 8 h (30°C). Interestingly, the *apm4*Δ cells did show some morphological change consistent with initiation of a projection (pointed cells); however, these cells did not proceed to form clear mating projections, suggesting polarity initiation but defective maintenance. In addition to an inability to form a mating projection, *apm4* null cells showed a greatly reduced sensitivity to alpha factor in halo assays (Figure S1, Supporting Information) possibly supporting the idea that morphological changes are required to maintain pheromone-induced growth arrest.

### Defects in polarity markers in apm4Δ cells

**Figure 3:**
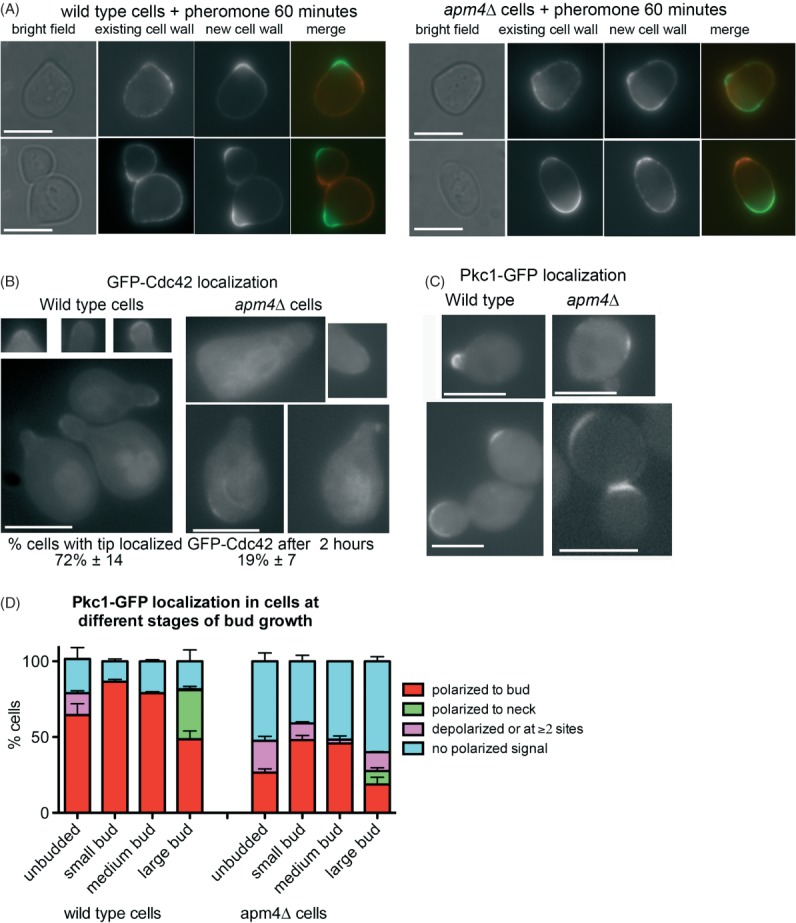
**Defects in polarity of cell wall markers in** ***apm4*****Δ cells.** A) Cells were incubated with concanavalin A conjugated to distinct fluorophores to label old and new growth of cells. Left panels show representative wild type cells; right panels show labelled *apm4* null cells. Scale bar = 5 µm. B) Wild type and *apm4*Δ cells were induced to express GFP-Cdc42 by growth in galactose containing medium for 3 h before addition of alpha factor for 2 h. Localization of GFP-Cdc42 was analysed in 100 cells in three independent experiments. Scale bar = 5 µm. C) Pkc1-GFP was analysed in cells to determine localization under conditions of vegetative growth. Scale bar = 5 µm. Shown are cells from different stages of budding. D) Localization of Pkc1-GFP was analysed in cells and extent of polarity recorded and shown graphically. Data are from three independent experiments with >100 cells counted in each experiment.

Having established that Apm4 is required for a polarized cell response to pheromone, the site of polarized growth was analysed further. Both plasma membrane and cell wall synthesis must be co-ordinated during growth, and cell wall deposition can be used to monitor where growth is occurring. Analysis using concanavalin A tagged with distinct fluorophores was used to follow cell wall growth prior to, and after pheromone addition. In wild type cells a focussed zone of new growth can be detected after pheromone addition (Figure 3A left panels). From analysis of 20 cells, the zone of growth was calculated to be between 4 and 8% of the cell surface. However, in the absence of Apm4, new cell wall growth could be observed over a broader region of the cell surface (up to 25% of the cell surface) and in some cells two separate sites of growth were observed suggesting that sites of polarity were either not being appropriately established, or that they could not be maintained (Figure 3A right panels).

Two small GTPases associated with polarity and cell wall deposition are Cdc42 and Rho1. Cdc42 binds to its GEF Cdc24 which is recruited to the site of polarity either a shmoo tip or site of bud emergence. In its active form Cdc42 is proposed to be required for establishing polarity through recruitment of actin polymerisation and secretory machinery 24. Cdc42 is considered to maintain its polarized location through two mechanisms one via its GDI removing it from the membrane and then facilitating its relocalization to the tip; the second being through an endocytic mechanism 25,26. GFP-Cdc42 was integrated, and expressed in cells in the presence and absence of *APM4*. In the presence of functional AP-2 the majority of cells showed a highly polarized localization of Cdc42 to the mating projection. This localization was significantly reduced in the *apm4* deletion strain.

A number of proteins have been shown to interact and localize with Rho1, a small GTPase required for activating the cell wall synthesis machinery including its GEF and the kinase Pkc1 27,28. Attempts were made to localize both GFP-tagged Rom2 (a Rho1 GEF) and Pkc1-GFP in cells in response to mating pheromone however, in both cases the presence of the tag appeared to render the cells non-responsive to alpha factor in otherwise wild type cells (data not shown). We therefore imaged cells in vegetative growth phase to determine whether absence of *apm4* impacted on Pkc1-GFP localization. As shown (Figure 3C), in wild type cells Pkc1-GFP localizes strongly to incipient bud sites and emerging buds with a slightly weaker signal at the plasma membrane of large buds, and also at sites of cytokinesis. In the absence of *apm4* about 50% of cells show little or no definable localization of Pkc1-GFP. In addition, some cells show two sites of localization, or localization at sites distinct from where a small bud has emerged. These data are shown graphically in Figure 3D. Taken together, the analysis of effects of *apm4* deletion indicate that it is required for polarity of markers to the tip of mating projections, and also for localization of cell wall machinery in vegetatively growing cells.

### Mid2 localization during polarized cell responses is AP-2 dependent

**Figure 4:**
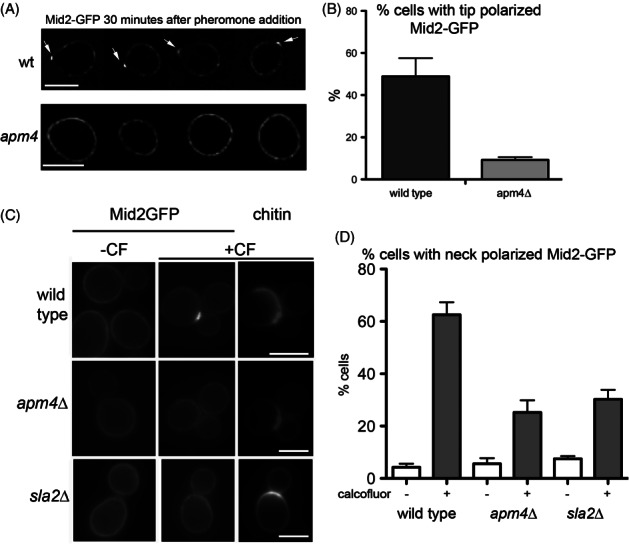
**Mid2 relocalization to the tip of mating projections and to the neck of calcofluor treated cells is reduced in** ***apm4*** **null cells.** A) Mid2-GFP localization was recorded after 30 min. Arrows indicate foci of Mid2-GFP localization in wild type cells. Scale bar = 5 µm. B) The proportion of cells showing Mid2-GFP at the tip was assessed in wild type and *apm4*Δ cells; *n* = 3 independent experiments. C) Mid2-GFP relocalizes to the mother bud neck region in wild type cells treated with calcofluor (CF). Left panels are Mid2-GFP in untreated cells, middle panels are Mid2-GFP in treated cells; right panels are the chitin staining by CF in the same treated cells. Scale bar = 5 µm. D) Analysis of Mid2 localization in the presence and absence of 150 µg/mL CF for 50 min in wild type, *apm4*Δ and *sla2*Δ cells. Data from three independent experiments. Errors bars are SEM. Unpaired *t*-test reveals significant reduction in Mid2-GFP relocalization in *apm4* Δ cells compared to wild type (p = 0.0007).

Mid2 is a cell wall stress sensor that is found to partially relocalize from a uniform plasma membrane distribution to the tip of the mating projection in the early stages of a pheromone response 29. It is proposed that in this location it functions to ensure that cell wall is generated appropriately during polarized cell growth. To determine whether Apm4 is involved in this process, cells expressing Mid2-GFP (integrated into the genome or plasmid-borne) were incubated with mating pheromone. Within 30 min Mid2 relocalization was clearly observed in wild type cells but deletion of *APM4* strongly inhibited this response suggesting that Apm4, as part of the AP-2 endocytic adaptor complex, functions to drive a portion of Mid2 from non-polarized plasma membrane sites to a focussed site at the tip of the mating projections (Figure 4A,B). If AP-2 is a *bona fide* endocytic adaptor for Mid2 it would also be predicted that an inability to internalize and recycle Mid2 in response to pheromone should lead to increased levels of Mid2-GFP at the membrane. Cells were analysed for surface levels of Mid2 following pheromone addition. Intensity of plasma membrane staining was significantly increased in the absence of Apm4 when compared to wild type controls strongly suggesting that Apm4 is responsible for Mid2 internalization and relocalization (Figure S2).

An additional polarized response of Mid2 is observed in log phase cells exposed to calcofluor. *In vivo*, calcofluor interacts specifically with chitin and induces synthesis of abnormal chitin fibrils 30. Chitin is enriched at the mother bud neck region in cells and is a critical component of the primary septum that forms during cytokinesis. Exposure to calcofluor causes a fraction of Mid2 to localize to the mother bud neck presumably responding to damage at this area enriched for chitin (Figure 4C). If AP-2 is an adaptor for Mid2 uptake in response to extracellular signals, it would be expected that defects in relocalization would also be observed in this situation. Cells expressing Mid2-GFP were exposed to calcofluor and the effect on Mid2-GFP localization monitored. Wild type, a*pm4Δ* and *sla2*Δ cells were analysed following calcofluor addition. *sla2*Δ cells have a severe endocytosis defect and were used to demonstrate the importance of endocytosis in this response. Quantification reveals a significant reduction in the number of cells showing a shift of Mid2-GFP to the bud neck (Figure 4D).

### Mid2 binds directly to the mu subunit of AP-2

**Figure 5:**
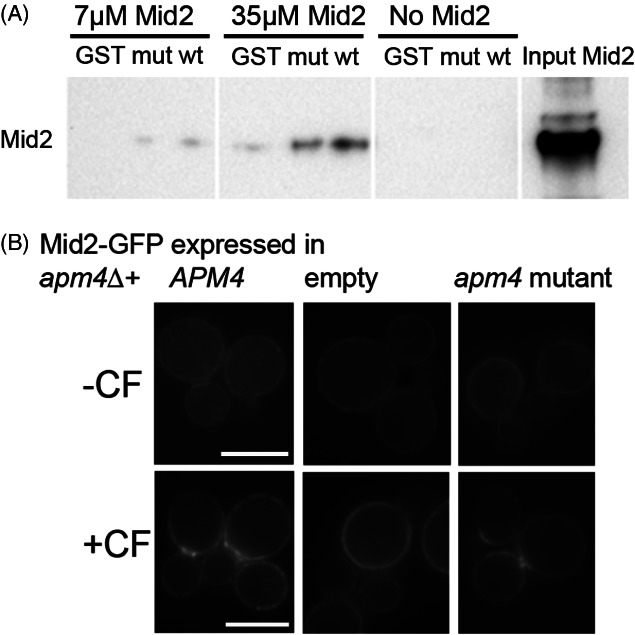
**Mid2 cytoplasmic tail interacts directly with the Apm4 mu homology region.** A) GST alone; GST tagged wild type Apm4 and GST tagged Apm4 mutants were prepared as described and incubated with His-tagged cytoplasmic tail of Mid2. Their binding interaction was tested using pull down assays on beads at two different concentrations of Mid2. Mid2 binding was detected using anti-His tag antibodies after western blotting. Input lane is 35 µm Mid2. B) Mid2-GFP was transformed into cells expressing wild type, mutant or no Apm4. Localization to the bud neck following 150 µg/mL CF was analysed. Scale bar = 5 µm.

In mammalian cells a number of cargoes, including the transferrin receptor, have been shown to bind directly to the mu homology domain of mu2 via a YXXΦ motif 31. To determine whether Mid2 is able to bind directly to Apm4, the cytoplasmic tail of Mid2 was expressed recombinantly as a His-tagged fusion 32 while the predicted cargo-binding mu homology domain of Apm4 was expressed as a GST fusion. Binding was tested using a GST-pull down assay. As shown (Figure 5A) Mid2 binds to Apm4-Ct but not to GST alone. Analysis of the AP-2 and AP-3 crystal structures reveals part of the mu subunit that interact with the YXXΦ motif 4,33. Three amino acids that show a substantial interaction with the YXXΦ motif reside near the C-terminus of the mu subunit. In yeast these residues are 476–480 KWIKY, corresponding to Human AP-2 mu subunit (Sequence Q96CW1) residues 420–424 of KWVRY. The central three residues of this motif were mutagenized to generate KAAAY, which would be predicted to lose the majority of interactions with the YXXΦ motif backbone. Purification of this mutant Apm4Ct still showed some binding to Mid2, but binding was judged from two independent experiments to be reduced by about 50%.

Given that mammalian mu2 subunit is able to bind some cargoes through a YXXΦ motif 31 it was noted that there is a single YXXΦ motif present in the cytoplasmic tail of Mid2 (residues 278–281) and the mutation Y278A was generated in the His-tagged Mid2 construct. This mutation again resulted also in a >50% reduction in binding compared to the wild type Mid2 tail indicating that the YXXΦ motif forms at least part of the Mid2, AP-2 binding site (Figure S3).

To test the requirement of the Apm4-Mid2 interaction for relocalization *in vivo*, the Apm4 WIK mutant (pKA1074) was expressed in cells and the effect of calcofluor addition analysed. When expressed in an *apm4*Δ *APL1-GFP* strain the mutant Apm4 was able to rescue Apl1-GFP localization to endocytic patches confirming that the protein was folding and interacting appropriately with other components of the AP2 complex (data not shown). Following calcofluor addition which led to Mid2GFP neck relocalization in wild type cells, this relocalization was also observed for this Apm4 mutant suggesting that the Apm4 interaction is not solely responsible for Mid2-GFP relocalization (Figure 5B). An analogous experiment was also performed to analyse the localization of the Mid2Y278A–GFP mutant compared to wild type Mid2-GFP in response to pheromone (Figure S4B). In response to pheromone, cells with Mid2-Y278A-GFP were still able to localize the sensor to the mating projection tip. 45 ± 12% showed tip localization compared with 49 ± 9% wild type Mid2-GFP localization (*n* = 3 experiments with > 50 cells each repeat; error is standard deviation).

## Discussion

The function of the mammalian AP-2 complex as an endocytic adaptor linking cargo molecules to the various endocytic coat proteins, including clathrin, has been well characterized. In contrast, studies in budding yeast have focussed on mechanistic aspects of membrane invagination and in particular on components present at endocytic sites that regulate the actin cytoskeleton 34. The identification of specific cargoes is relatively less well studied and, with the exception of ubiquitin moieties and the presence of NPFXD motifs, relatively little is known about cargoes or how they are recognized for internalization 35–38. In this study we aimed to determine conditions requiring functionality of AP-2 and to identify potential binding partners of the complex. The most striking findings are that AP-2 complex function is required for maintaining morphological changes triggered during the pheromone response, and that during this response the relocalization of Mid2 requires a functional AP-2 complex. Thus, the AP-2 complex in *S. cerevisiae* appears to carry out a function very similar to that observed in mammalian cells in endocytic cargo uptake. A function in regulating vesicle coat formation however, seems less likely as the appendage domains which make multiple interactions with other coat components in mammalian AP-2 are only present on the yeast alpha subunit (Apl3) and no defects in general endocytic uptake are observed in the absence of AP-2 12.

*In vitro* analysis reveals a binding interaction between the C-terminal region of Apm4 and the cytoplasmic tail of Mid2. The level of binding is relatively low, similar to that obtained for other endocytic cargo-adaptor interactions 4,6,42. Mutation of either the likely YXXΦ motif binding site in Apm4, or the YXXΦ motif in Mid2 itself, both reduced binding between Apm4 and Mid2 cytoplasmic region *in vitro*, demonstrating that the observed interaction between the proteins is indeed disrupted by the mutations as predicted. The lack of a phenotype when the mutations are expressed *in vivo* suggests the presence of other possible motifs including dileucine motifs (also in the Mid2 tail) which would bind the sigma subunit, or indeed other as yet uncharacterized motifs.

A number of mathematical models have been generated and tested focussing on generation and maintenance of cell polarity using *S. cerevisiae* as a model system. In these, endocytosis has been proposed to be of central importance in keeping Cdc42 polarized to ensure an appropriate growth pattern 25,26,39. Other studies have also indicated the importance of endocytosis to limit the functional zone of cell wall deposition to ensure it occurs only within the active growth zone 20. It has been suggested that corralling the growth site with regions of high levels of endocytosis will ensure that appropriate polarity markers are internalized and recycled 39. However, in our analysis of *apm4*Δ cells, there does not appear to be a significant change in the organization of endocytic patches during vegetative growth or in response to pheromone when compared to wild type cells (Figure S4). This suggests that additional layers of regulation function to ensure some specificity in the endocytosis that takes place within the endocytic zone.

While endocytosis is known to be required for polarity, the proteins mediating uptake of key polarity factors are not known. In this study we investigated localization of the stress sensor Mid2. This protein was previously found to partially relocalize from a uniform plasma membrane distribution to the tip of the mating projection in the early stages of a pheromone response 29 and its tip localization was observed even in the absence of de novo biosynthesis. In addition, after photobleaching, Mid2 shmoo tip accumulation recovered suggesting that for pheromone addition (though not yet demonstrated for calcofluor addition), tip localization was not due to polarized exocytosis 29. Here we show that Mid2 is a cargo for AP-2, and that in the absence of AP-2 it is not relocalized in response to pheromone or to cell wall damage by calcofluor. Mid2 was originally identified in a screen for mutations that died on exposure to mating pheromone 40. Coupled with its identification as a cell wall stress sensor and as a protein that binds and activates cell wall machinery 41, we consider that in order to undergo the rapid growth necessary at the site of mating projection growth, a portion of the cell wall sensing machinery is relocated to the appropriate site. It seems unlikely however that Mid2 is the only cargo being internalized in this polarity response and future studies will aim to identify further AP-2 cargoes in the proteome. However, it is interesting to note that this study adds to the complexity of internalization motifs known for the family of cell wall stress receptors with the receptors Wsc1 and Wsc2 using NPFXD motifs and ubiquitin, respectively 36–38.

Notably, the cytoplasmic tail of Mid2 has also been shown to bind to the endocytic site protein Syp1 32. This protein does not have a conventional mu adaptin homology domain but it does carry a related muniscin mu homology domain. It was considered that these two proteins might act redundantly in binding Mid2, however, a double deletion of *apm4*Δ*syp1*Δ did not exacerbate any of the observed phenotypes (data not shown).

A critical question remaining is what triggers the AP-2-Mid2 interaction, as Mid2 during vegetative growth is evenly distributed over the cell surface. Regulation of mammalian AP-2 cargo binding is proposed to occur by phosphorylation of a residue T156 lying in a hinge region between the N terminal longin and C-terminal mu adaptin homology domain 8,42. Mutation of this phosphorylation site precludes cargo uptake *in vivo*, while dephosphorylation in a Rab5 dependent manner is important for AP-2 release and vesicle uncoating 10,43. These data suggest that while phospholipid binding might open up the AP-2 complex for possible interactions, these interactions might only occur *in vivo* in response to additional signalling events. The availability of online datasets of phosphorylated peptides in the *S. cerevisiae* proteome reveal that phosphorylation can indeed take place in the same hinge region of Apm4 as seen for mammalian mu2 (www.phosphogrid.org). Indeed 11 residues lying between S157 and S188 (10 serines, 1 threonine) have been found to be phosphorylated. Of these, specific conditions inducing phosphorylation have been reported for residues S179, S180 and S181 which have all been found to be phosphorylated in a cell cycle dependent manner 44. In addition, S180 is phosphorylated in a manner dependent on the high osmolarity (HOG) MAPK pathway 45. Taken together, our data, coupled with phospho-proteome analysis suggests a model in which a signal, for example pheromone addition, activates a response pathway leading to Apm4 phosphorylation and activation or stabilization of its cargo-binding site. AP-2 is then able to bind certain cargoes, facilitate their internalization and allow them to be trafficked to appropriate polarized sites.

## Materials and Methods

### Materials

Unless stated otherwise, chemicals were obtained from Sigma-Aldrich. Media was from Melford Laboratories, Ipswich, Suffolk, UK (yeast extract, peptone and agar) or Sigma (minimal synthetic medium and amino acids).

### Yeast strains, plasmids and cell growth

Yeast strains used in this study are listed in Table S1. Plasmids used were pKA982 (pGEX6P1+-Apm4 amino acids 179–491); pKA989 (*APM4* nucleotides minus 301 to1476); pJJH1557 (Mid2-GFP, *LEU2*, *CEN*); pKA1042 as pJJH982+Mid2 Y278A); pET28 URA, KAN, Mid2 aa251-376; pKA983 (Mid2 251-376 Y278A); pRWC151 (GFP-Cdc42 LEU). The Apm4 477-479 WIK-AAA mutations was made in pKA989 generating pKA1074; the Y278A point mutation in the *MID2* gene was generated using a QuikChange Lightning site directed mutagenesis kit (Stratagene) with plasmids pET28 + Mid2(251-376) and pJJH982 as templates and the point mutations. The *apm4* and sla2 deletions were generated using PCR integration methodology 46. *S. cerevisiae* cells were grown with rotary shaking at 30°C in liquid YPD medium (1% yeast extract, 2% Bacto-peptone, 2% glucose supplemented with 40 µg/mL adenine) or in synthetic medium (0.67% yeast nitrogen base, 2% glucose) with appropriate supplements. For experiments using pheromone, alpha factor was purchased from Sigma and used at 2 µg/mL to induce mating projection formation. GFP-Cdc42 was induced by growth for 3 h in SD medium containing 2% galactose instead of glucose. Calcofluor was added at 150 µg/mL. Low ammonia medium (SLAD) for pseudohyphal growth in diploids was prepared (6.7 g/L yeast nitrogen base without amino acids and ammonium sulphate, 2% glucose, 2% washed Bacto-agar and appropriate auxotrophic requirements). Strains scored for pseudohyphal filament formation were streaked on SLAD plates and observed and imaged after 1 week of growth at 30°C. *C. albicans* cultures were grown on YEPD (2% glucose, 2% Difco Bacto-peptone and 1% Difco Bacto yeast extract) plus 80 mg/L uridine. Hyphal growth was promoted in liquid culture by adding 20% calf serum (Sigma-Aldrich, St Louis), adjusting the pH to 7.0 and incubating at 37°C 47 or by growth on plates containing Spider medium [1.35% agar, 1% nutrient broth, 0.4% potassium phosphate and 2% mannitol (pH 7.2)] 48. Gene deletions were performed as previously described 49. Transformations were performed using lithium acetate as previously described 50.

### Biochemical approaches

His-tagged Mid2 cytoplasmic tail and GST-tagged Apm4 were expressed in *Escherichia coli* (BL21(DE3)) after 5 h induction with IPTG at 30°C, and purified using His-trap columns (GE Healthcare) and Glutathione sepharose resin (GE Healthcare), respectively. For the binding assay, glutathione sepharose beads, with GST-tagged Apm4 or the Apm4 mutant still bound, were blocked overnight in buffer containing 50 mm NaCl, 20 mm sodium phosphate buffer pH 7.5, 1 mm MgCl2, 5% BSA and 10% glycerol. Control beads were also prepared and blocked similarly using GST only. Recombinant Mid2 was incubated with the Apm4-bound beads for 30 min in the same buffer, and unbound protein was washed away with 3× 1 mL washes of the same buffer. Proteins bound to Apm4 were separated on a 15% SDS PAGE gel and Mid2 was detected by western blotting using anti-His antibody.

### Fluorescence methods and microscopy

To visualize the old and new growth of cell wall, Alexa488 and Alexa568 concanavalin A were used as described 51.

Epifluorescence microscopy was performed using an IX-81 inverted microscope (Olympus) with a Cool Snap HQ2 cooled CCD camera (Photometrics), and Image ProPlus image capture software; Media Cybernetics. Images were exported as TIFF files and assembled using Adobe Photoshop cs2. Statistical analysis of localization was performed using Graphpad Prism software.

## Supporting Information

**Table S1:** Yeast strains used in this study.

**Figure S1: Halo assays to demonstrate effect of pheromone on growth arrest in wild type and apm4 null strains.** KAY120 and KAY1690 were grown to lawns in the presence of discs supplemented with 1, 2 and 5 µg α-factor. Cells were grown on YPD as described.

**Figure S2: Mid2-GFP levels at the plasma membrane are higher in *apm4* null cells.** After 30 min of exposure to pheromone the fluorescence intensity of plasma membrane staining was analysed in wild type and *apm4*Δ cells as a measure of the level of Mid2-GFP. Intensities were measured at two points per cell where a line drawn perpendicular to the axis of polarized growth assessed morphologically, and through the centre of the cell, bisected the plasma membrane. Data from three independent experiments and analysis of >100 cells total. In a Student *t*-test significance p value <0.0001. Mean wt 380.1 ± 14.24 SEM, *n* = 103; *apm4*Δ mean 637.9 ± 25.73, *n* = 103.

**Figure S3: The effect on Apm4 binding and Mid2 localization of a Y278 mutation in the Mid2 cytoplasmic tail.** A) GST alone; GST-tagged Apm4; His-tagged Mid2 or Mid2 Y278 were prepared as described and their binding interaction tested using pull down assays on beads with 20 µm Mid2. Apm4 is clearly seen on a Generon NUView gel, along with a faint band for Mid2 (arrow). Mid2 was detected using anti-His tag antibodies after western blotting. This band was verified as Mid2 using mass spectrometry. B) Mid2-GFP and Mid2-Y278A-GFP were transformed into cells otherwise lacking *mid2* (KAY53) or both *mid2* and *apm4* (KAY1700). Localization after 1 h incubation with alpha factor was observed microscopically.

**Figure S4: Effect of pheromone on organization of actin patches in wild type and apm4Δ cells.** Abp1-GFP was transformed into KAY120 and KAY1690 cells and visualized in live cells 90 min after pheromone addition.
