# Supplementary material for: Yeast Endocytic Adaptor AP-2 Binds the Stress Sensor Mid2 and Functions in Polarized Cell Responses
Source: Traffic. 2014 Feb 25;15(5):546–57. doi: 10.1111/tra.12155 (PMC4282331; doi:10.1111/tra.12155)
Supplement: Supplementary file 1 — Table S1: Yeast strains used in this study. [file tra0015-0546-SD1.pdf]

Table 1: Yeast strains used in this study

Name	Genotype	Notes/origin
BWP17	*URA3*::λ*imm434/URA3*::λ*imm434his1*::*hisG/his1*::*hisG* *arg4*::*hisG/arg4*::*hisG*	Wilson 1999
KAY1776	*URA3*::λ*imm434/URA3*::λ*imm434his1*::*hisG/his1*::*hisG* *arg4*::*hisG/arg4*::*hisG*, *apm4*Δ::*ARG4/apm4*Δ::*URA3*	This study
KAY1217	**a**/α *his3*Δ*/his3*Δ, *leu2*Δ*/ leu2*Δ, *ura3*Δ*/ ura3*Δ (Σ1278 strain)	D.Drubin, Berkeley
KAY1732	**a**/α *his3*Δ*/his3*Δ, *leu2*Δ*/ leu2*Δ, *ura3*Δ*/ ura3*Δ, *apm4*Δ::*HIS3/apm4*Δ::*HIS3*	This study
BY4741	*MAT***a** *his3*Δ*1, leu2*Δ*, ura3*Δ*, met15*Δ	Invitrogen
KAY1742	*MAT***a** *his3*Δ*1, leu2*Δ*, ura3*Δ*, met15*Δ*, APL1-GFP::HIS3*	This study
KAY1747	*MAT***a** *his3*Δ*1, leu2*Δ*, ura3*Δ*, met15*Δ*, APL1-GFP::HIS3 apm4*Δ*::KanMx*	This study
KAY736	*MAT***a** *his3*Δ*1, leu2*Δ*, ura3*Δ*, met15*Δ*, MID2-GFP::HIS3*	Invitrogen
KAY1733	*MAT***a** *his3*Δ*1, leu2*Δ*, ura3*Δ*, met15*Δ*, MID2-GFP::HIS3, apm4*Δ*::URA3*	This study
KAY53	*MATa glc7*Δ*LEU2, trp1::GLC7::TRP1, ade2-1, his3-11, leu2-3,112, trp1-1, ura3-1can1-100, ssd1-d2, Gal+, mid2*Δ*::URA3*	This study
KAY120	*MAT***a** *ura3-52, lys2-801, ade2-101, trp1-*Δ*63, his3-*Δ*200, leu2-*Δ*1, sst1::hisG*	J.Thorner (Berkeley)
KAY1690	*MAT***a** *ura3-52, lys2-801, ade2-101, trp1-*Δ*63, his3-*Δ*200, leu2-*Δ*1, sst1::hisG, apm4*Δ*::URA3*	This study
KAY1700	*MAT***a** *his3*Δ*1, leu2*Δ*, ura3*Δ*, met15*Δ*, apm4*Δ*::KanMx*	This study
KAY1798	KAY120 with integrated *GFPCdc4::LEU2*	This study
KAY1799	KAY1690 with integrated *GFPCdc4::LEU2*	This study
KAY1805	KAY736 + *sla2*Δ*LEU2*	This study
KAY1787	*MAT***a** *ade2-1 leu2-3,112 his3-11,15 trp1-1 ura3-1 PKC1-GFP(S65T)::His3MX can1-100 ssd1-d2* Gal^+	(27)
KAY1788	KAY1787 + *apm4*Δ::*URA3*	This study
