# Supplementary material for: Yeast Endocytic Adaptor AP-2 Binds the Stress Sensor Mid2 and Functions in Polarized Cell Responses
Source: Traffic. 2014 Feb 25;15(5):546–57. doi: 10.1111/tra.12155 (PMC4282331; doi:10.1111/tra.12155)

Supplementary Figure 1
Halo assays to demonstrate effect of pheromone on growth arrest in wild type and apm4 null strains. KAY120 and KAY1690 were grown to lawns in the presence of discs supplemented with 1,2 and 5 µg α-factor. Cells were grown on YPD as described.

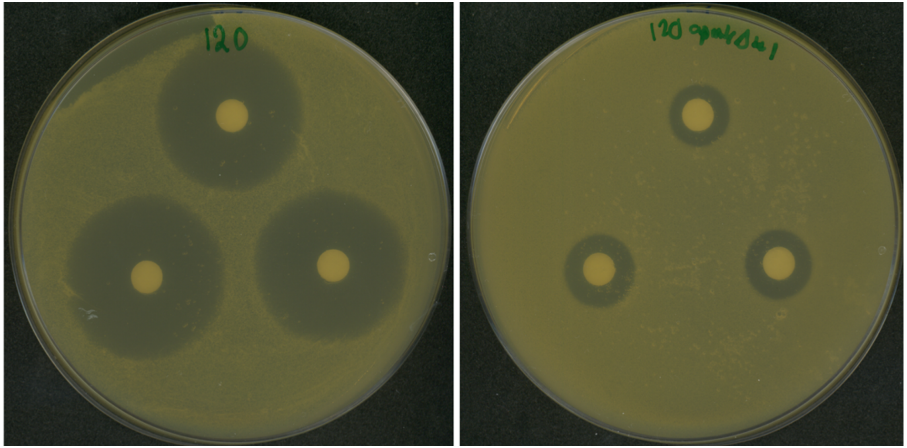

Supplement: Supplementary file 3 — Figure S1: Halo assays to demonstrate effect of pheromone on growth arrest in wild type and apm4 null strains. KAY120 and KAY1690 were grown to lawns in the presence of discs supplemented with 1, 2 and 5 µg α-factor. Cells were grown on YPD as described. [file tra0015-0546-SD3.pdf]
