# Supplementary material for: Yeast Endocytic Adaptor AP-2 Binds the Stress Sensor Mid2 and Functions in Polarized Cell Responses
Source: Traffic. 2014 Feb 25;15(5):546–57. doi: 10.1111/tra.12155 (PMC4282331; doi:10.1111/tra.12155)

Supplementary Figure 2

Mid2-GFP levels at the plasma membrane are higher in *apm4* null cells. After 30 minutes of exposure to pheromone the fluorescence intensity of plasma membrane staining was analysed in wild type and *apm4*Δ cells as a measure of the level of Mid2-GFP. Intensities were measured at 2 points per cell where a line drawn perpendicular to the axis of polarised growth assessed morphologically, and through the centre of the cell, bisected the plasma membrane. Data from 3 independent experiments and analysis of > 100 cells total. In a student t-test significance P value <0.0001. Mean wt 380.1 ± 14.24 SEM n=103; *apm4*Δ mean 637.9 ±25.73 n=103.

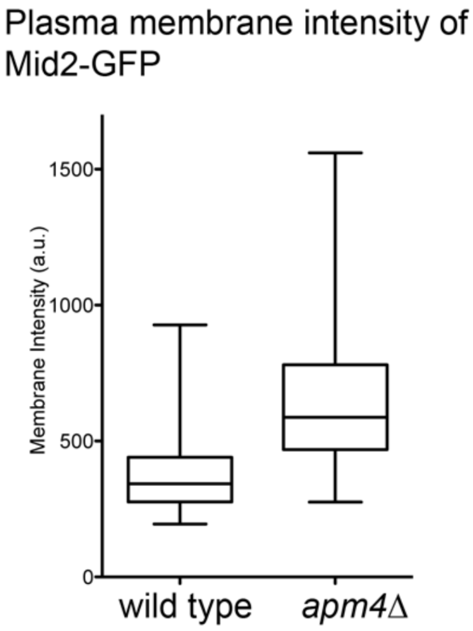

Supplement: Supplementary file 5 — Figure S2: Mid2-GFP levels at the plasma membrane are higher in apm4 null cells. After 30 min of exposure to pheromone the fluorescence intensity of plasma membrane staining was analysed in wild type and apm4Δ cells as a measure of the level of Mid2-GFP. Intensities were measured at two points per cell where a line drawn perpendicular to the axis of polarized growth assessed morphologically, and through the centre of the cell, bisected the plasma membrane. Data from three independent experiments and analysis of >100 cells total. In a Student t-test significance p value <0.0001. Mean wt 380.1 ± 14.24 SEM, n = 103; apm4Δ mean 637.9 ± 25.73, n = 103. [file tra0015-0546-SD5.pdf]
