# Supplementary material for: Yeast Endocytic Adaptor AP-2 Binds the Stress Sensor Mid2 and Functions in Polarized Cell Responses
Source: Traffic. 2014 Feb 25;15(5):546–57. doi: 10.1111/tra.12155 (PMC4282331; doi:10.1111/tra.12155)

Supplementary Figure 3. **The effect on Apm4 binding and Mid2 localization of a Y278 mutation in the Mid2 cytoplasmic tail.** (A) GST alone; GST tagged Apm4 ; His-tagged Mid2 or Mid2 Y278 were prepared as described and their binding interaction tested using pull down assays on beads with 20 µM Mid2. Apm4 is clearly seen on a Generon NUView gel, along with a faint band for Mid2 (arrow). Mid2 was detected using anti-His tag antibodies after western blotting. This band was verified as Mid2 using mass spectrometry. (B) Mid2-GFP and Mid2-Y278A-GFP were transformed into cells otherwise lacking *mid2* (KAY53) or both *mid2* and *apm4* (KAY1700). Localization after 1 hour incubation with alpha factor was observed microscopically.

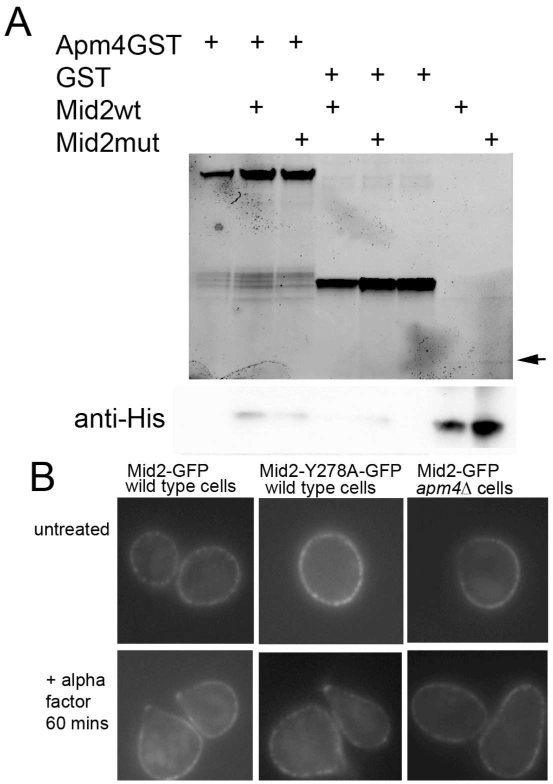

Supplement: Supplementary file 7 — Figure S3: The effect on Apm4 binding and Mid2 localization of a Y278 mutation in the Mid2 cytoplasmic tail. A) GST alone; GST-tagged Apm4; His-tagged Mid2 or Mid2 Y278 were prepared as described and their binding interaction tested using pull down assays on beads with 20 µm Mid2. Apm4 is clearly seen on a Generon NUView gel, along with a faint band for Mid2 (arrow). Mid2 was detected using anti-His tag antibodies after western blotting. This band was verified as Mid2 using mass spectrometry. B) Mid2-GFP and Mid2-Y278A-GFP were transformed into cells otherwise lacking mid2 (KAY53) or both mid2 and apm4 (KAY1700). Localization after 1 h incubation with alpha factor was observed microscopically. [file tra0015-0546-SD7.pdf]
