# Supplementary material for: Yeast Endocytic Adaptor AP-2 Binds the Stress Sensor Mid2 and Functions in Polarized Cell Responses
Source: Traffic. 2014 Feb 25;15(5):546–57. doi: 10.1111/tra.12155 (PMC4282331; doi:10.1111/tra.12155)

**Supplementary Figure 3**

**Effect of pheromone on organization of actin patches in wild type and apm4Δ cells**. Abp1-GFP was transformed into KAY120 and KAY1690 cells and visualized in live cells 90 minutes after pheromone addition.

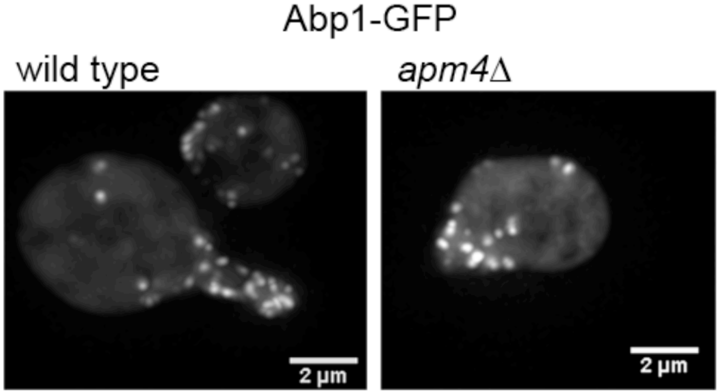

Supplement: Supplementary file 9 — Figure S4: Effect of pheromone on organization of actin patches in wild type and apm4Δ cells. Abp1-GFP was transformed into KAY120 and KAY1690 cells and visualized in live cells 90 min after pheromone addition. [file tra0015-0546-SD9.pdf]
