# Supplementary material for: Yeast Endocytic Adaptor AP-2 Binds the Stress Sensor Mid2 and Functions in Polarized Cell Responses
Source: Traffic. 2014 Feb 25;15(5):546–57. doi: 10.1111/tra.12155 (PMC4282331; doi:10.1111/tra.12155)

Supplementary Materials and Methods

Halo assay to assess α-factor sensitivity

This method is used to qualitatively assess the α-factor sensitivity of your **a** strain of interest and relies on the production of a zone of growth inhibition in a lawn of yeast cells by a range of concentrations of α-factor present on small discs of filter paper. Grow up 3 ml cultures of strains in appropriate medium overnight to about 1-5 x 10^7cells/ml. Count cells and calculate the volume required to give 1 x 10^6cells total. Melt 1% sterile agar and cool to <55°C. Dilute appropriate volume of the culture into 2 ml of 2 x YPD. Add 2 ml molten agar, mix gently and pour onto the surface of a YPD plate. Move the plate gently to ensure the agar covers the surface. Leave to solidify (a few minutes). Prepare α-factor dilutions to give 1 µg, 2.5 µg, 5 µg, each in 10 µl volume. Place sterile antibiotic assay discs on surface of lawn and pipette α -factor onto discs and incubate for 1-2 days, until a lawn of cells has grown and halos are visible. Overall halo diameters can be measured and the radius plotted against $\log_{10}$ (amount of α-factor). This should give straight lines. The displacement of these lines can be used to estimate relative sensitivity.

Localization of actin structures in wild type and *apm4*Δ cells. In order to visualize organization and appearance of actin structures initial attempts were made to use rhodamine phalloidin staining. While this gave good staining in both strains in vegetative growth, after pheromone addition *apm4*Δcells did not give any clear staining pattern, rather a diffuse rhodamine stain was observed (data not shown). To address whether this was due to mislocalization of actin or due to an effect on the phalloidin binding site, cells were transformed with a reporter for actin patch localization, Abp1-GFP. Abp1 localized to patches in both strains and did not become mislocalized on pheromone addition indicating that a change in the availability of the phalloidin binding site occurred in the *apm4*Δ cells following pheromone addition. The reason for this is unclear but was observed with 2 independently isolated *apm4*Δ strains. In the cells expressing Abp1-GFP, a similar number of endocytic patches were visualized in the different strains. Clear polarization of patches to the tip region was observed in the wild type strain, however even in the *apm4*Δ mutant, localization to the more ‘pointed’ end of cells was observed indicating that lack of polarity is not due to an inability to polarize actin patches per se.

Table 1: Yeast strains used in this study

Name	Genotype	Notes/origin
BWP17	*URA3*::λ*imm434/URA3*::λ*imm434his1*::*hisG/his1*::*hisG* *arg4*::*hisG/arg4*::*hisG*	Wilson 1999
KAY1776	*URA3*::λ*imm434/URA3*::λ*imm434his1*::*hisG/his1*::*hisG* *arg4*::*hisG/arg4*::*hisG*, *apm4*Δ::*ARG4/apm4*Δ::*URA3*	This study
KAY1217	**a**/α *his3*Δ*/his3*Δ, *leu2*Δ*/ leu2*Δ, *ura3*Δ*/ ura3*Δ (Σ1278 strain)	D.Drubin, Berkeley
KAY1732	**a**/α *his3*Δ*/his3*Δ, *leu2*Δ*/ leu2*Δ, *ura3*Δ*/ ura3*Δ, *apm4*Δ::*HIS3/apm4*Δ::*HIS3*	This study
BY4741	*MAT***a** *his3*Δ*1, leu2*Δ*, ura3*Δ*, met15*Δ	Invitrogen
KAY1742	*MAT***a** *his3*Δ*1, leu2*Δ*, ura3*Δ*, met15*Δ*, APL1-GFP::HIS3*	This study
KAY1747	*MAT***a** *his3*Δ*1, leu2*Δ*, ura3*Δ*, met15*Δ*, APL1-GFP::HIS3 apm4*Δ*::KanMx*	This study
KAY736	*MAT***a** *his3*Δ*1, leu2*Δ*, ura3*Δ*, met15*Δ*, MID2-GFP::HIS3*	Invitrogen
KAY1733	*MAT***a** *his3*Δ*1, leu2*Δ*, ura3*Δ*, met15*Δ*, MID2-GFP::HIS3, apm4*Δ*::URA3*	This study
KAY53	*MATa glc7*Δ*LEU2, trp1::GLC7::TRP1, ade2-1, his3-11, leu2-3,112, trp1-1, ura3-1can1-100, ssd1-d2, Gal+, mid2*Δ*::URA3*	This study
KAY120	*MAT***a** *ura3-52, lys2-801, ade2-101, trp1-*Δ*63, his3-*Δ*200, leu2-*Δ*1, sst1::hisG*	J.Thorner (Berkeley)
KAY1690	*MAT***a** *ura3-52, lys2-801, ade2-101, trp1-*Δ*63, his3-*Δ*200, leu2-*Δ*1, sst1::hisG, apm4*Δ*::URA3*	This study
KAY1700	*MAT***a** *his3*Δ*1, leu2*Δ*, ura3*Δ*, met15*Δ*, apm4*Δ*::KanMx*	This study
KAY1798	KAY120 with integrated *GFPCdc4::LEU2*	This study
KAY1799	KAY1690 with integrated *GFPCdc4::LEU2*	This study
KAY1805	KAY736 + *sla2*Δ*LEU2*	This study
KAY1787	*MAT***a** *ade2-1 leu2-3,112 his3-11,15 trp1-1 ura3-1 PKC1-GFP(S65T)::His3MX can1-100 ssd1-d2* Gal^+	(27)
KAY1788	KAY1787 + *apm4*Δ::*URA3*	This study

Supplementary Figure 1
Halo assays to demonstrate effect of pheromone on growth arrest in wild type and apm4 null strains. KAY120 and KAY1690 were grown to lawns in the presence of discs supplemented with 1,2 and 5 µg α-factor. Cells were grown on YPD as described.

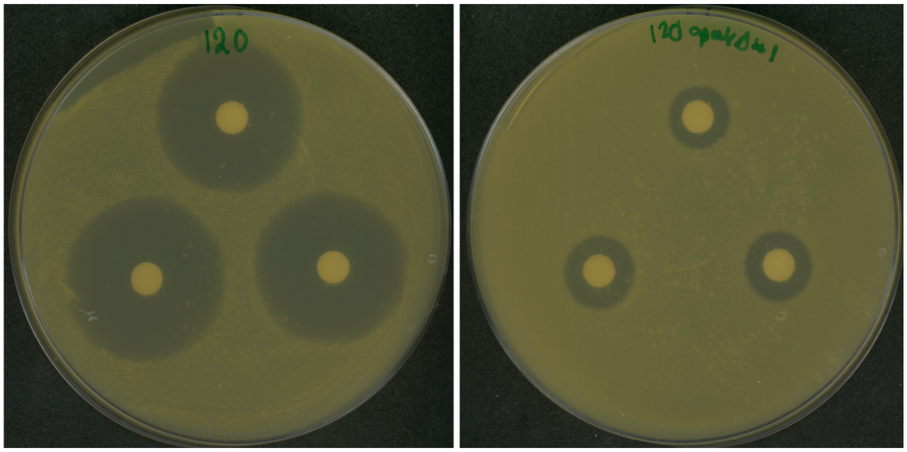

Supplementary Figure 2
Mid2-GFP levels at the plasma membrane are higher in *apm4* null cells. After 30 minutes of exposure to pheromone the fluorescence intensity of plasma membrane staining was analysed in wild type and *apm4*Δ cells as a measure of the level of Mid2-GFP. Intensities were measured at 2 points per cell where a line drawn perpendicular to the axis of polarised growth assessed morphologically, and through the centre of the cell, bisected the plasma membrane. Data from 3 independent experiments and analysis of > 100 cells total. In a student t-test significance P value <0.0001. Mean wt 380.1 ± 14.24 SEM n=103; *apm4*Δ mean 637.9 ±25.73 n=103.

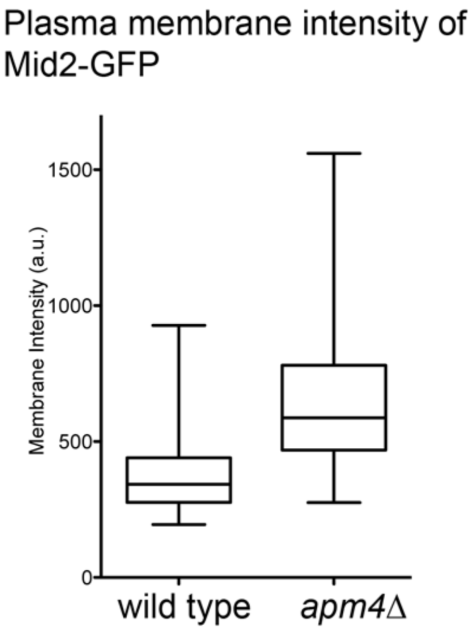

Supplementary Figure 3. **The effect on Apm4 binding and Mid2 localization of a Y278 mutation in the Mid2 cytoplasmic tail.** (A) GST alone; GST tagged Apm4 ; His-tagged Mid2 or Mid2 Y278 were prepared as described and their binding interaction tested using pull down assays on beads with 20 μM Mid2. Apm4 is clearly seen on a Generon NUView gel, along with a faint band for Mid2 (arrow). Mid2 was detected using anti-His tag antibodies after western blotting. This band was verified as Mid2 using mass spectrometry. (B) Mid2-GFP and Mid2-Y278A-GFP were transformed into cells otherwise lacking *mid2* (KAY53) or both *mid2* and *apm4* (KAY1700). Localization after 1 hour incubation with alpha factor was observed microscopically.

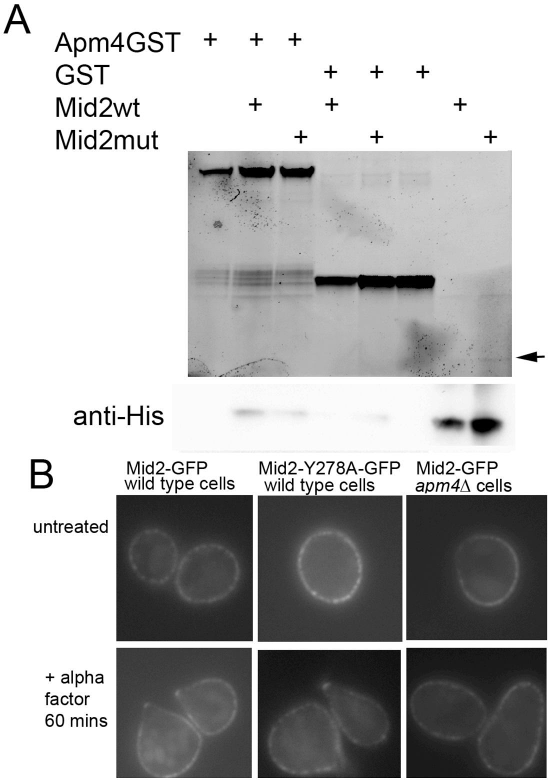

Supplementary Figure 3
Effect of pheromone on organization of actin patches in wild type and apm4Δ cells. Abp1-GFP was transformed into KAY120 and KAY1690 cells and visualized in live cells 90 minutes after pheromone addition.

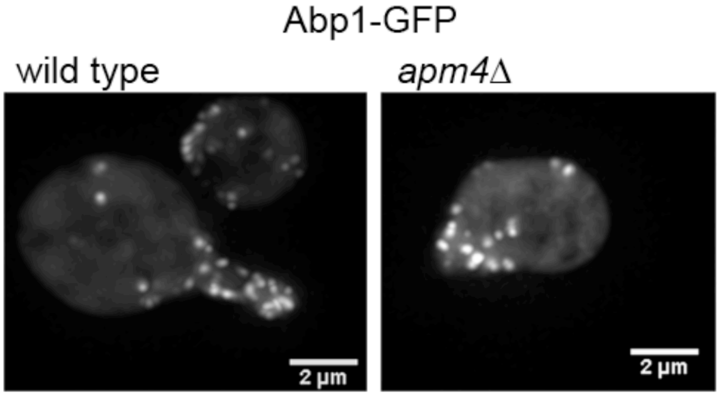

Supplement: Supplementary file 11 — Appendix S1. Supplementary materials and methods. [file tra0015-0546-SD11.pdf]
